# Randomized phase II study evaluating a carbon ion boost applied after combined radiochemotherapy with temozolomide versus a proton boost after radiochemotherapy with temozolomide in patients with primary glioblastoma: The CLEOPATRA Trial

**DOI:** 10.1186/1471-2407-10-478

**Published:** 2010-09-06

**Authors:** Stephanie E Combs, Meinhard Kieser, Stefan Rieken, Daniel Habermehl, Oliver Jäkel, Thomas Haberer, Anna Nikoghosyan, Renate Haselmann, Andreas Unterberg, Wolfgang Wick, Jürgen Debus

**Affiliations:** 1 Department of Radiation Oncology, University Hospital of Heidelberg, Im Neuenheimer Feld 400, 69120 Heidelberg, Germany; 2 Institute of Medical Biometry and Informatics, University of Heidelberg, Im Neuenheimer Feld 305, 69120 Heidelberg, Germany; 3 Heidelberger Ionenstrahl Therapiezentrum (HIT), Im Neuenheimer Feld 450, 69120 Heidelberg, Germany; 4 Department of Neurosurgery, University Hospital of Heidelberg, Im Neuenheimer Feld 400, 69120 Heidelberg, Germany; 5 Department of Neurooncology, University Hospital of Heidelberg, Im Neuenheimer Feld 400, 69120 Heidelberg, Germany

## Abstract

**Background:**

Treatment standard for patients with primary glioblastoma (GBM) is combined radiochemotherapy with temozolomide (TMZ). Radiation is delivered up to a total dose of 60 Gy using photons. Using this treatment regimen, overall survival could be extended significantly however, median overall survival is still only about 15 months.

Carbon ions offer physical and biological advantages. Due to their inverted dose profile and the high local dose deposition within the Bragg peak precise dose application and sparing of normal tissue is possible. Moreover, in comparison to photons, carbon ions offer an increase relative biological effectiveness (RBE), which can be calculated between 2 and 5 depending on the GBM cell line as well as the endpoint analyzed. Protons, however, offer an RBE which is comparable to photons.

First Japanese Data on the evaluation of carbon ion radiation therapy showed promising results in a small and heterogeneous patient collective.

**Methods/Design:**

In the current Phase II-CLEOPATRA-Study a carbon ion boost will be compared to a proton boost applied to the macroscopic tumor after surgery at primary diagnosis in patients with GBM applied after standard radiochemotherapy with TMZ up to 50 Gy. In the experimental arm, a carbon ion boost will be applied to the macroscopic tumor up to a total dose of 18 Gy E in 6 fractions at a single dose of 3 Gy E. In the standard arm, a proton boost will be applied up to a total dose 10 Gy E in 5 single fractions of 2 Gy E.

Primary endpoint is overall survival, secondary objectives are progression-free survival, toxicity and safety.

**Discussion:**

The Cleopatra Trial is the first study to evaluate the effect of carbon ion radiotherapy within multimodality treatment of primary glioblastoma in a randomized trial comparing this innovative treatment of the treatment standard, consisitng of photon radiotherapy in combination with temozolomide.

**Trial Registration:**

ISRCTN37428883 and NCT01165671

## Background

Glioblastomas (GBM) are the most common primary brain tumors in adults; they are characterized by a rapid and infiltrative growth pattern. In spite of extensive research over the past years, outcome of patients with GBM still remains unsatisfactory.

With surgery and supportive care alone, overall survival is about 3-5 months. Postoperative radiotherapy (RT) can increase overall survival to 9-12 months [1]. A number of studies have shown that an additional treatment with chemotherapy can increase overall survival. This benefit, however, is commonly associated with a high risk of treatment-related side effects, especially with combination treatments, such as PCV, and with carmustine (BCNU). Only recently, significant increase in overall survival could be achieved by adding temozolomide (TMZ), an orally applicable alkylating substance, to postoperative radiotherapy. In a prospective randomized Phase III study performed by the EORTC radiochemotherapy with TMZ was compared to postoperative radiation alone. Overall survival could be increased from 12.1 months to 14.6 months, with acceptable toxicity. TMZ was applied in a dose of 75mg/m^2^/die during radiotherapy, followed by 6 cycles of adjuvant TMZ [2]. Therefore, standard treatment of patients with GBM is currently considered to be postoperative radiochemotherapy with TMZ, followed by 6 cycles of adjuvant TMZ.

However, with an overall survival of about 15 months, treatment outcome still remains unsatisfactory. Therefore, a number of treatment concepts are currently under investigation.

Novel radiotherapeutic modalities such as carbon ion radiotherapy offer a promising treatment alternative. Radiation therapy using charged particles is characterized by distinct physical and biological characteristics. Charged particles provide the physical advantage of an inverted dose profile which enables steep dose gradients. Neighbouring organs at risk can be spared much better. Heavy charged particles, such as carbon ions, additionally offer an increased relative biological effectiveness (RBE).

Carbon ion radiotherapy was available by the Department of Radiation Oncology at the Gesellschaft für Schwerionenforschung (GSI) in Darmstadt since 1997. Superior treatment results for a number of tumor entities, such as chordomas and chondrosarcomas of the skull base, as well as adenoid cystic carcinomas (ACC) have been shown, and carbon ion radiotherapy is currently performed in the clinical routine for these patients [3-6]. Safety of carbon ion radiotherapy with respect to critical organs at risk, such as the brain, brainstem or spinal chord, have been shown in these studies. At the Heidelberg Ion Therapy Center (HIT), treatment of over 1300 patients per year with Proton and Carbon ion RT is possible.

In general, GBM are treatment-resistant tumors. Early studies using a high-dose proton boost could show that total doses up to 90 Gy E were effective in preventing local tumor recurrences, however, such high doses were associated with high rates of side effects [7].

In vitro data for the treatment of GBM with carbon ions have shown superior effectivity compared to photons [8]. Our own data have shown a high RBE for carbon ion RT for GBM; additionally combination of carbon ion radiotherapy and TMZ have been evaluated and show an additive effect in GBM-cell lines [9]. A first clinical study evaluating a carbon ion boost in patients with GBM was recently published by Mizoe et al. [10]. Median overall survival in patients with glioblastoma was 17 months; however, only small patient numbers were evaluated and standard chemotherapy with TMZ was not applied. In that study, the carbon ion boost was applied with stepwise increasing total doses up to 24.8 Gy E. While toxity was low even in the high dose arm, the data showed that patients seem to benefit from the high dosed carbon ion boost. Therefore, the concept of a carbon ion boost to patients with GBM with a macroscopic tumor lesion after neurosurgical resection is a promising treatment alternative and requires evaluation in a larger patient group with GBM. Additionally, the carbon ion boost should be evaluated in combination with standard radiochemotherapy with TMZ.

In the present CLEOPATRA trial, the impact of a carbon ion boost will be compared to a proton boost using intensity-modulated rasterscanning in patients with incompletely resected GBM in combination with standard radiochemotherapy with TMZ. TMZ will be continued during the standard and experimental arm as prescribed in standard photon radiochemotherapy.

Protons offer a comparable RBE to photons. A number of pre-clinical and clinical studies have shown that the effect of proton radiotherapy on normal tissue as well as on tumors is comparable to photon radiotherapy. The RBE values for clinical proton beams have been determined for a wide spectrum of *in vitro *as well as *in vivo *systems, as well as in clinical trials [11,12]. Therefore, it has been concluded that proton therapy can replace photon therapy without any further clinical trials when the same dose is applied [13]. In the current study, the proton dose applied is equivalent to the standard dose applied in conventional radiochemotherapy with photons. Moreover, protons and carbon ions are both characterized by the same physical characteristics, i.e. Bragg Peak with a high local dose deposition. This results in a reduction on dose to normal tissue in proton and carbon ion radiotherapy compared to conventional photon radiotherapy. These physical characteristics can be considered comparable for the proton and the carbon ion beam, and both are able to convey to improved dose distributions as compared to photons. Thus, reduction of side effects on normal tissue can be achieved by particle therapy (proton or carbon ion) compared to conventional photon radiotherapy. The aim of the present study however is not to evaluate the distinct physical characteristics, therefore it is essential that treatment techniques and physical characteristics are identical in both treatment arms. The aim of this study is solely to compare the effect of the increased RBE in the carbon ion arm on outcome. Therefore, the proton treatment arm is considered to be the standard treatment arm, and carbon ion radiotherapy is the experimental treatment arm.

## Methods and Design

### Study design

The purpose of the trial is to compare a carbon ion boost to a proton boost delivered to the macroscopic tumor in combination with combined radiochemotherapy with TMZ in patients with primary GBM.

The aim of the study is to compare overall survival as a primary endpoint, and progression free survival, toxicity and safety as secondary endpoints.

Focus of the analysis is to evaluate the change in overall survival and local control by carbon ion radiotherapy. Therefore, the aim of the trial is to evaluate the improvement in outcome due to effect of the altered biology of carbon ions on GBM. Chemotherapy with TMZ is considered standard treatment and is administered continuously as it would be applied in standard patient care outside any trial.

### Trial Design

**Figure 1:**
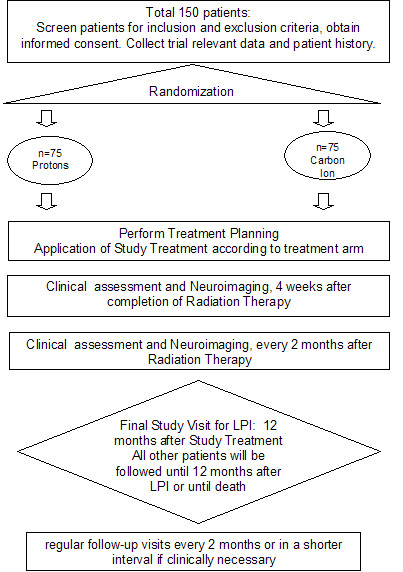
**Workflow diagram of the Cleopatra Study evaluation the role of carbon ion radiotherapy for the treatment of patients with primary glioblastoma**.

The trial will be performed as a single-center two-armed randomized Phase II study. The trial workflow and treatment arms are depicted in Fig. 1.

Patients fulfilling the inclusion criteria will be randomized into two arms:

Arm A - Experimental Arm

Carbon Ion Radiation Therapy as a Boost to the macroscopic tumor

Total Dose 18 Gy E, 6 fractions, 3 Gy E single dose

Arm B - Standard Arm

Proton Radiation Therapy as a Boost to the macroscopic tumor

Total Dose 10 Gy E, 5 fractions, 2 Gy E single dose

Standard chemotherapy with TMZ will be continued during the experimental and standard arm in conventional dosing of 75 mg/m^2 ^per day.

### Study objectives

The primary objective is overall survival during the follow-up phase of at least 12 months (starting with initial diagnosis).

The secondary objectives of the study are progression-free survival, toxicity and safety.

### Patient selection: Inclusion criteria

Patients meeting all of the following criteria will be considered for admission to the trial:

- histologically confirmed unifocal, supratentorial primary glioblastoma

- macroscopic tumor after biopsy or subtotal resection

- indication for combined radiochemotherapy with temozolomide

- prior photon irradiation of 48-52 Gy to the T2-hyperintense area, resection cavity, areas of contrast enhancement adding 2-3 cm safety margin in combination with standard temozolomide

- registration prior to photon RT or within photon RT allowing the beginning of particle therapy ≤4 days after completion of photon irradiation

- beginning of study treatment (proton or carbon ion RT) no later than 12 weeks after primary diagnosis

- age ≥18 years

- Karnofsky Performance Score ≥60

- adequate contraception.

- Ability of subject to understand character and individual consequences of the clinical trial

- Written informed consent (must be available before enrolment in the trial)

### Patient selection: Exclusion criteria

Patients presenting with any of the following criteria will not be included in the trial:

- refusal of the patients to take part in the study

- previous radiotherapy of the brain or chemotherapy with DTIC or TMZ other than during the radiochemotherapy stated in the inclusion criteria

- more than 52 Gy applied via photon-RT prior to particle therapy

- time interval of >12 weeks after primary diagnosis (neurosurgical intervention) and beginning of study treatment (proton or carbon ion RT)

- Patients who have not yet recovered from acute toxicities of prior therapies

- Clinically active kidney, liver or cardiac disease

- Known carcinoma <5 years ago (excluding Carcinoma in situ of the cervix, basal cell carcinoma, squamous cell carcinoma of the skin) requiring immediate treatment interfering with study therapy

- Pregnant or lactating women

- Participation in another clinical study or observation period of competing trials, respectively.

### Treatment Assignment

Radiation therapy according to the protocol will be performed in patients included into the study and after assignment of the patients to the treatment arms after randomization. Patients withdrawn from the trial retain their identification codes (e.g. randomization number, if already given). New patients must always be allotted a new identification code.

### Treatment Planning

For particle therapy, patients will be immobilized using an individually manufactured head mask. For treatment planning, contrast-enhanced CT as well as MR-imaging will be performed for optimal target definition.

Patients included to the study will have received 48-52 Gy of photon RT applied to the resection cavity, area of contrast enhancement on T1-weighted MR-Imaging, T2-weighted hyperintensities including a safety margin of 2-3 cm.

Treatment planning for carbon ion and proton radiotherapy should be performed during photon radiotherapy about 1-2 weeks prior to the start of the carbon ion or proton boost.

Organs at risk such as the brain stem, optic nerves, chiasm and spinal chord will be contoured. Dose constraints of normal tissue will be respected according to Emami et al. [14]. The Gross Tumor Volume (GTV) will be defined for the proton or carbon ion boost as the area of contrast enhancement on T1-weighted MR-imaging; for the Clinical target Volume (CTV), a safety margin of 5 mm is added.

Amino-Acid-PET or SPECT-Examinations may be used in addition to contrast-enhanced MRI for target volume definition but are not mandatory.

Carbon ion RT planning is performed using the treatment planning software PT-Planning (Siemens, Erlangen, Germany) including biologic plan optimization. Biologically effective dose distributions will be calculated using the a/β ratio for GBM as well as for the endpoint late toxicity to the brain.

No interruptions >4 days between the end of photon radiotherapy and study treatment (carbon ion or proton boost) are allowed.

Patient positioning prior to particle therapy will be evaluated by comparison of x-rays to the DRRs. Set up deviations >3 mm will be corrected prior to radiotherapy.

### Dose Prescription Experimental (Carbon) Arm

The intensity-controlled rasterscan system will be used for beam application. Six fractions of a single dose of 3 Gy E up to a total dose of 18 Gy E will be prescribed to the maximum of the calculated dose distribution for the target volume (Boost). Treatment planning aims in the coverage of the target volume by the 90%-isodose line.

Dose specification is based on biologic equivalent dose because of the high relative biologic effectiveness (RBE) of carbon ions, which differs throughout the target volume due to its dependence on various factors. RBE will be calculated at each voxel throughout the target volumes and biological optimization will be performed. The dose prescription used is related to the isoeffective dose Gy E (Cobalt Gray equivalent) using daily fractions of 2 Gy and a weekly fractionation of 5 × 2 Gy.

### Dose Prescription Standard (Proton) Arm

The intensity-controlled rasterscan system will be used for proton beam application. Five fractions of a single dose of 2 Gy E up to a total dose of 10 Gy E will be prescribed to the maximum of the calculated dose distribution for the target volume (Boost). Treatment planning aims in the coverage of the target volume by the 90%-isodose line.

For proton beams, an RBE of about 1.1 can be considered. Therefore, the 10 Gy E applied with proton radiotherapy after treatment of 50 Gy with photons prior to study inclusion add up to the standard dose of 60 Gy used in conventional chemo-radiation with TMZ.

### Follow-up

The primary endpoint is overall survival at 12 months, therefore patients are followed within the trial protocol for a minimum 12 months after completion of study treatment. For the LPI, the final study visit will be 12 months after study treatment to assess the primary endpoint. All other patients will be followed on a regular basis as stated below until death or until 12 months after LPI.

After RT, patients are scheduled for follow-up visits every 2 months or as needed clinically including contrast-enhanced MRI as well as thorough clinical-neurological assessment.

The last patient included into the study will be followed 12 months after treatment. This is considered the final study visit. All other patients will be followed regularly as described in detail until death or until 12 months after LPI.

If death occurs <12 months or patients leave the study prior to12 months, they will be still included into the ITT population.

The overall duration of the trial is expected to be approximately 48 months. Recruitment of the patients is planned over a time period of 36 months, minimum duration of the follow-up phase will be 12 months. An interim analysis is planned after 50% of the expected events have occurred.

### Statistical calculations for trial sample size

The calculation of sample size for the CLEOPATRA trial is based on the analysis of the primary endpoint 'time to death for any reason during the follow-up phase of at least 12 months (starting with initial diagnosis)' using the logrank test to compare the survival curves of the experimental and the standard treatment. The specific assumptions and methods underlying this calculation are explained below.

#### 1. Recruitment period, follow-up duration and allocation ratio

The recruitment period will last 36 months with constant accrual. The minimum follow-up time (for the last patient included in the trial if no event occurs before) will be 12 months, the maximum follow-up time (for the first patient included in the trial if no event occurs before) will be 48 months. Patients will be randomized to the treatment groups with an equal allocation ratio of 1:1.

#### 2. Overall survival rate

Stupp et al. [2] investigated a study population very similar to the one expected in the CLEOPATRA trial. Hence, according to their results the one-year survival rate for the standard treatment is expected to be about 60%.

#### 3. Treatment effect size

The CLEOPATRA trial will be designed to detect an improvement of 20% (absolute) in survival rate after 12 months. The rationale behind this assumption is as follows: Carbon ion radiotherapy is characterized by a higher relative biological effectiveness (RBE) as compared to conventional photons or protons. Preclinical experiments on glioblastoma cell lines have shown that this RBE lies between 3 and 5, depending on the respective endpoint or cell line [9]. Therefore, the effect of carbon ion radiotherapy for the treatment of patients with primary glioblastoma is expected to be substantially higher than with conventional proton radiotherapy.

#### 4. Type I error rate and power

Time to death for any reason will be compared between the two treatment groups using a two-tailed logrank test stratified for RPA class at an overall type I error rate of 5%. The desired power is 90%. Sample size calculation is based on the unstratified logrank test. It can be expected that including the RPA class (which has a major prognostic impact on survival [2]) as a covariate will actually increase the power as compared to the unstratified test. For this reason, the sample size resulting from our calculations should assure the desired power of 90% under the assumptions made.

#### 5. Interim analysis

An interim analysis will be performed according to the stopping rule of O'Brien-Fleming published in 1979 after 50% of the expected events have been occurred [15].

#### 6. Methods used for sample size calculation

For sample size calculation, exponentially distributed survival times are assumed to calculate the hazard ratio under the alternative. Th*e *formulae proposed by Schoenfeld (which hold true under the less restrictive assumption of proportional hazards) were used [16,17]. Calculations were done using the software ADDPLAN 4.0 [18].

Using the assumptions enumerated above, the resulting total sample size yielding the necessary number of events is 150 patients (75 per treatment group). The expected total number of events under the null-hypothesis is then about 136, under the alternative-hypothesis it amounts to about 115. The total sample size of 150 patients will provide a power of 90,7% to detect a 20% (absolute) improvement in 12 months overall survival rate of the experimental treatment compared to the standard treatment. With this target sample size, CLEOPATRA will also have an adequate power to detect a smaller than anticipated treatment effect (more than 78% power to detect an improvement of 17% (absolute) in 12 months overall survival rate).

### Statistical Methods

#### Confirmatory Analysis

A confirmatory analysis with characteristics common for studies designed for the proof of efficacy (two-sided test problem with significance level 5%) is implemented in this Phase II study in order to be able to show superiority of the experimental treatment as compared to the standard treatment in case of a significant result. Confirmatory analysis for the primary endpoint 'time to death for any reason during the follow-up phase of at least 12 months (starting with initial diagnosis)' is based on the full analysis set which is defined according to the intention-to-treat principle and includes all randomized patients. The null-hypothesis of equal survival curves for the two treatment groups is tested by applying the two-sided logrank test stratified by *RPA class. The overall type I error rate is 5%. A g*roup-sequential design is applied with one interim analysis after half of the expected number of events has occurred. The stopping rule is according to [15]: The one-sided critical level in the interim analysis is given by 0.26%, and in the final analysis it is given by 2.4%; the null-hypothesis can be rejected, if the smaller of the two one-sided p-values falls below one of these boundaries. Withdrawals, lost of follow-ups and patients who are still alive by the end of the follow-up phase are treated as censored observations. The censoring date is given by the last known date at which the patient is still alive. The survival curves will be estimated using the Kaplan-Meier product-limit method, and the corresponding confidence intervals will be calculated using Greenwood's formula [19].

#### Descriptive Analyses

To assess the impact of major protocol deviations, an analogous analysis of the primary outcome variable will be performed for the per protocol set of all patients without major protocol violations. A descriptive analysis of the primary outcome variable is performed applying a Cox-regression model including the covariates RPA class, age, Karnofsky index and extent of previous surgery.

Analysis of the secondary endpoint 'progression-free survival' will be performed analogously to the primary endpoint whereby the p-values of the logrank test and the tests within the Cox-regression model will be interpreted descriptively. All further documented variables will be analyzed descriptively by tabulation of the measures of the empirical distributions. Descriptive p-values of the corresponding statistical tests comparing the treatment groups and associated 95% confidence intervals will be given. The homogeneity of the treatment groups will be described by comparison of the demographic data and the baseline values of the measured variables.

Safety analysis and analysis of toxicity will be based on the data set of all randomized patients who were treated with the experimental or the standard treatment at least once. The safety analysis includes calculation and comparison of frequencies and rates of adverse and serious adverse events reported in the two treatment groups.

All analyses will be done using SAS version 9.1 or higher (for the handling of survival analyses with SAS see, [20]).

#### Interim Analysis

To allow for early stopping in case of an overwhelming treatment effect, a group-sequential design is applied with one interim analysis that is performed when 50% of the number of events expected under the null-hypothesis have occurred. Under the assumptions made in sample size calculation, the interim analysis is performed after occurrence of 68 events which should happen approximately 26 months after start of recruitment [18]. The stopping rule is specified according to O'Brien and Fleming (1979; [15]).

No formal boundary for stopping for futility is specified. However, if the results of the interim analysis suggest that the objectives of the study cannot be reached with a feasible number of patients or that the benefit/risk ratio for the study has worsened markedly, the study may be stopped by decision of the principle investigator. As in this case the null-hypothesis would not be rejected, no type I error would be committed and therefore the type I error rate of the study would still be controlled at 5%.

### Data Handling, Storage and Archiving of Date

All findings including clinical and laboratory data will be documented by the investigator or an authorized member of the study team in the subject's medical record and in the CRF. The investigator is responsible for ensuring that all sections of the CRF are completed correctly and that entries can be verified against source data. In some cases, the CRF, or part of the CRF, may also serve as source documents: Karnofsky Performance Status, Documentation of Clinical-Neurological Examination.

Data will be collected by the Study Center at the Department of Radiation Oncology, University Hospital of Heidelberg, Heidelberg, Germany.

After receipt of the CRF-pages by the principal investigator of the study, all data will be entered in a study specific database as recorded in the CRF.

All missing data or inconsistencies will be reported back to the investigators and clarified by the responsible investigator. If no further corrections are to be made in the database it will be declared closed and used for statistical analysis.

The data will be stored and archived according to §13 of the German GCP-Regulation and §28c of the German X-Ray Regulation (RöV) and §87 of the German Radiation Protection Regulation (StrlSchV) for at least 30 years after the trial termination.

### Ethics, informed consent and safety

A positive Ethics Vote was obtained by the Local Ethics Committee of the medical Faculty at the University of Heidelberg, Germany.

Additionally, a positive vote of the Bundesamt für Strahlenschutz (BfS) has been obtained.

### Treatment at tumor progression

After completion of study treatment (5 fractions of proton therapy or 6 fractions of carbon ion radiotherapy according to the treatment arms of the CLEOPATRA trial), adjuvant cycles of chemotherapy with TMZ are recommended in conventional dosing according to the Stupp regimen [2]. Any systemic treatment or chemotherapy is not part of the clinical trial.

For tumor progression, treatment alternatives will be evaluated and discussed interdisciplinary considering options of neurosurgical resection, systemic treatment such as chemotherapy, a second course of radiation therapy, or other.

## Discussion

Interdisciplinary treatment of patients with GBM still remains a challenge in spite of major advances over recent years. The addition of alkylating chemotherapy with the advent of TMZ has significantly increased progression-free and overall survival, however, with a median survival of about 15 months, treatment optimization remains a major challenge [2,21,22]. For more than two decades clinical studies have shown that RT is the single most effective treatment for GBM; however, even after application of high local doses, the majority of recurrences develops within the high-dose irradiation field [23,24]. Therefore, dose-escalation studies have been performed, but increase in dose was mostly related with a steep increase in severe treatment-related side effects.

In contrast to the typical photon dose deposition curve, particle therapy is characterized by a low dose deposition within the entry channel of the particle beam, and the steep dose deposition called the Bragg peak that can be directed by variation of beam energy directly into the defined target volume. Thereafter, a steep dose fall-off helps sparing of normal tissue behind the treatment volume. These physical advantages contribute significantly to the clinical advantage of particle therapy, enabling the radiation oncologist to increase sparing of normal tissue, and subsequently to deliver higher doses to the defined target volume. Carbon ion radiotherapy is additionally characterized by an increased relative biological effectiveness (RBE) due to severe radiation damage produced by the carbon beam. This higher RBE has been shown to translate into improved tumor control rates, especially for treatment-resistant indications.

In the past, particle therapy was only available in few centers worldwide, Today, several institutions offer proton radiotherapy within clinical routine, however, carbon ions are only available in few centers in Japan and Germany. The Department of Radiation Oncology at the University Hospital of Heidelberg is offering carbon ion radiotherapy within clinical routine since 1997. Since then, over 450 patients have been treated successfully with the carbon beam at the Gesellschaft für Schwerionenforschung (GSI) in Darmstadt, Germany. Within the clinical studies performed, excellent clinical results were reported for chordomas and chondrosarcomas of the skull base, high-risk meningiomas as well as adenoid-cystic carcinomas [5,6,25-27]. For several other indications the superiority of particle beams have been demonstrated as summarized by Schulz-Ertner and Tsujii [28].

In Japan, patients with malignant gliomas have been treated with carbon ion beams with convincing results. In a study published by Mizoe et al. patients with anaplastic astrocytomas as well as GBM were treated with photon radiotherapy and a carbon ion boost to the macroscopic tumor. Median overall survival for GBM patients was 17 months, and even the highest doses of carbon ions applied at 24.8 Gy E were tolerated without any severe treatment-related side effects [10]. The increased RBE of carbon ions have been proven by preclinical studies showing an increase in effectivity between 2 and 5, depending on the cell line analyzed [8,9,29]. Today, the standard treatment approach for GBM after neurosurgical resection is combined radiochemotherapy with TMZ as described above [2,22]. *In vitro*, an additive effect of carbon ion radiotherapy and TMZ has been demonstrated [9].

Therefore, In the current Phase II-CLEOPATRA-Study a carbon ion boost will be compared to a proton boost applied to the macroscopic tumor after surgery at primary diagnosis in patients with GBM in combination with after standard radiochemotherapy with TMZ. The study will offer the possibility to compare the carbon ion boost in combination with standard chemotherapy to standard treatment due to the randomized nature of this trial. The aim of the trial is to target the region most likely of tumor recurrence, i.e. the macroscopic region of the tumor within the high-dose irradiation field, and to combine this novel approach with established treatment standards.

## Competing interests

The authors declare that they have no competing interests.

## Authors' contributions

SEC, JD, MK, WW, AU and AN have developed the study concept. SEC, JD and MK wrote the study protocol and obtained ethics approval. SEC, SR, DH, JD, WW, AU, AN and RH will provide patient care. TH and OJ will perform treatment planning and beam application for carbon ion radiotherapy. SEC, WW, JD, and RH will implement the protocol and oversee collection of the data. All authors contributed to and approved the final manuscript.

## Pre-publication history

The pre-publication history for this paper can be accessed here:

http://www.biomedcentral.com/1471-2407/10/478/prepub
